# The role of aircraft noise annoyance and noise sensitivity in the association between aircraft noise levels and medication use: results of a pooled-analysis from seven European countries

**DOI:** 10.1186/s12889-021-10280-3

**Published:** 2021-02-05

**Authors:** Clémence Baudin, Marie Lefèvre, Wolfgang Babisch, Ennio Cadum, Patricia Champelovier, Konstantina Dimakopoulou, Danny Houthuijs, Jacques Lambert, Bernard Laumon, Göran Pershagen, Stephen Stansfeld, Venetia Velonaki, Anna L. Hansell, Anne-Sophie Evrard

**Affiliations:** 1 Univ Lyon, Univ Gustave Eiffel, IFSTTAR, Univ Lyon 1, Umrestte, UMR T_9405, Bron, France; 2 Now at: Institute for Radiological Protection and Nuclear Safety, Fontenay-aux-Roses, France; 3 Now at: Technical Agency for Information on Hospital Care, Lyon, France; 4 Currently retired (formerly Federal Environment Agency), Berlin, Germany; 5 Environmental Health Unit, Agency for Health Protection, Pavia, Italy; 6 Univ Gustave Eiffel, IFSTTAR, AME-DCM, Bron, France; 7 Department of Hygiene, Epidemiology and Medical Statistics, Medical School, National and Kapodistrian University of Athens, Athens, Greece; 8 National Institute for Public Health and the Environment, Bilthoven, the Netherlands; 9 Currently retired, Villeurbanne, France; 10 Univ Gustave Eiffel, IFSTTAR, TS2, Bron, France; 11 Institute of Environmental Medicine, Karolinska Institute, Stockholm, Sweden; 12 Centre for Psychiatry, Wolfson Institute of Preventive Medicine, Barts and the London School of Medicine, Queen Mary University of London, London, UK; 13 Nurses School, National and Kapodistrian University of Athens, Athens, Greece; 14 Centre for Environmental Health and Sustainability, University of Leicester, Leicester, UK

**Keywords:** Aircraft noise exposure, Aircraft noise annoyance, Noise sensitivity, Medication use

## Abstract

**Background:**

Few studies have considered aircraft noise annoyance and noise sensitivity in analyses of the health effects of aircraft noise, especially in relation to medication use. This study aims to investigate the moderating and mediating role of these two factors in the relationship between aircraft noise levels and medication use among 5860 residents of ten European airports included in the HYENA and DEBATS studies.

**Methods:**

Information on aircraft noise annoyance, noise sensitivity, medication use, and demographic, socio-economic and lifestyle factors was collected during a face-to-face interview at home. Medication was coded according to the Anatomical Therapeutic Chemical (ATC) classification. Outdoor aircraft noise exposure was estimated by linking the participant’s home address to noise contours using Geographical Information Systems (GIS) methods. Logistic regressions with adjustment for potential confounding factors were used. In addition, Baron and Kenny’s recommendations were followed to investigate the moderating and mediating effects of aircraft noise annoyance and noise sensitivity.

**Results:**

A significant association was found between aircraft noise levels at night and antihypertensive medication only in the UK (OR = 1.43, 95%CI 1.19–1.73 for a 10 dB(A)-increase in L_night_). No association was found with other medications. Aircraft noise annoyance was significantly associated with the use of antihypertensive medication (OR = 1.33, 95%CI 1.14–1.56), anxiolytics (OR = 1.48, 95%CI 1.08–2.05), hypnotics and sedatives (OR = 1.60, 95%CI 1.07–2.39), and antasthmatics (OR = 1.44, 95%CI 1.07–1.96), with no difference between countries. Noise sensitivity was significantly associated with almost all medications, with the exception of the use of antasthmatics, showing an increase in ORs with the level of noise sensitivity, with differences in ORs among countries only for the use of antihypertensive medication. The results also suggested a mediating role of aircraft noise annoyance and a modifying role of both aircraft noise annoyance and noise sensitivity in the association between aircraft noise levels and medication use.

**Conclusions:**

The present study is consistent with the results of the small number of studies available to date suggesting that both aircraft noise annoyance and noise sensitivity should be taken into account in analyses of the health effects of exposure to aircraft noise.

**Supplementary Information:**

The online version contains supplementary material available at 10.1186/s12889-021-10280-3.

## Introduction

Over the years, many studies have shown that exposure to aircraft noise has adverse effects on the health of people living near airports: annoyance [1], sleep disturbance [2, 3], cardiovascular disease including hypertension [4–7], altered cognitive performance in children [8, 9], and disruption in hormonal rhythm [10–12]. Psychological disorders could also be considered as possible negative effects of aircraft noise exposure, but the role of aircraft noise annoyance and noise sensitivity in this relationship remains unclear [13].

Medication use has been shown to be a more objective and reliable measure of health outcomes than health measures based on self-reported symptoms alone [14, 15]. A review of the literature has indicated that when medication use is available, it should be considered a feasible (and perhaps preferable) indicator of environmental health [16]. A small number of studies have focused on the relationship between aircraft noise levels and medication use by airport residents, suggesting an association with the use of antihypertensive medication, and possibly psychotropic drugs or sleep medication [17–20].

Noise has been found to activate the sympathetic and endocrine system, which defines it as a psychosocial stressor. Babisch introduced a noise response model [21–23] indicating that the mechanism of noise effects would involve a direct pathway through synaptic interactions and an indirect pathway through cognitive perception of sound, including annoyance and noise sensitivity. Both pathways involve sleep disturbance that can lead to physiological stress reactions, resulting in adverse effects such as cardiovascular disease (hypertension in particular), and psychological disorders that could have negative consequences on cardiovascular function [24].

The very large number of studies that have investigated the association between noise exposure and noise annoyance showed that aircraft noise is, for a given level of noise exposure, the most annoying of all transportation noise sources [25, 26]. Significant associations between aircraft noise annoyance and psychological distress or deterioration of mental health [13, 27–30] and of general health (based on self-reported symptoms) [31, 32], have been observed. The association between aircraft noise annoyance and medication use has been investigated in only a few studies, suggesting that the use of antihypertensives, anxiolytics or hypnotics, and psychotropic drugs increased significantly with increasing aircraft noise annoyance [17, 33]. The coexistence of the two associations, between noise levels and annoyance due to noise on the one hand, and between annoyance due to noise and health effects on the other hand, could suggest a mediating role played by noise annoyance in the relationship between noise levels and adverse health effects. This role has been assumed in particular in studies on psychological disorders or mental health [27, 34, 35]. In addition, it has been stated that aircraft noise annoyance may have a modifying role in the relationship between aircraft noise levels and hypertension risk [36–38]: this association would be higher in highly annoyed participants.

The association between noise sensitivity and adverse effects of noise has also been little studied, but the findings are consistent: noise sensitivity has been found to be associated with increased blood pressure [39], health complaints (including cardiac complaints) [31, 40, 41], hypertension, psychological distress [40] and the use of psychotropic drugs (sleeping pills, tranquilizers and analgesics) [42, 43]. In addition, some studies have found stronger associations between noise exposure and some adverse health effects in highly sensitive individuals: a modifying effect of noise sensitivity has been suggested in the association between aircraft noise levels and anxiety and nervous complaints [31], psychological disorders [44, 45], heart rate [46], hypertension [38] and self-reported physical health problems [29, 31, 47].

While aircraft noise annoyance and noise sensitivity have been shown to affect the associations between aircraft noise exposure and different health effects, their role in the relationship between aircraft noise levels and medication use has never been investigated and therefore deserves further research.

In this study, the larger number of participants obtained by pooling data from two major European studies on aircraft noise and health that used similar methodology, HYENA (HYpertension and Exposure to Noise near Airports) and DEBATS (Discussion on the health effects of aircraft noise) provided additional power to explore interactions and offered for the first time the opportunity to investigate the impact of aircraft noise annoyance and noise sensitivity on medication use, and their modifying and mediating role on the relationship between aircraft noise levels and medication use in such a large study population. For this purpose, the same methodology as that followed in the article by Baudin and colleagues on the risk of hypertension was used [38].

## Methods

### Study population

The HYENA cross-sectional study was conducted between 2004 and 2006 and included 4861 participants (2404 men and 2457 women) aged 45–70 at the time of the interview and living near some of Europe’s busiest airports: London Heathrow (United Kingdom), Berlin Tegel (Germany), Amsterdam Schiphol (the Netherlands), City Airport Bromma and Stockholm Arlanda (Sweden), Milan Malpensa (Italy), and Athens Elephterios Venizelos (Greece) airports [48]. Registration offices, electoral rolls, and health services were used to randomly select participants.

The DEBATS cross-sectional study was conducted in 2013 and included 1244 participants (549 men and 695 women) older than 18 years of age at the time of the interview and living near some of France’s and Europe’s busiest airports: Paris-Charles de Gaulle, Lyon-Saint-Exupéry, and Toulouse-Blagnac [5]. A phone directory with addresses in the study area was used to randomly select participants.

Both HYENA and DEBATS participants signed and returned an informed consent to participate in the study.

In both studies, a face-to-face questionnaire was used to collect demographic and socio-economic characteristics; smoking habits, alcohol consumption, physical activity and other lifestyle factors; personal medical history and medication use; sleep disturbance, noise annoyance, and noise sensitivity (see supplementary files ‘HYENA Questionnaire.doc’ and ‘DEBATS Questionnaire.doc’). In addition, blood pressure (BP) and anthropometric measurements (weight and height) were taken.

### Aircraft noise exposure assessment

For all countries, except the UK where the national Aircraft Noise Contour Model (ANCON v 2) was used [49], the “Integrated Noise Model” (INM) was used to estimate outdoor aircraft noise levels in intervals of 1 dB(A) at the place of residence of the participants in front of the buildings [50]. The home address of each participant was linked to the noise contours using Geographical Information Systems (GIS) methods. For the statistical analyses in this study, we focused on aircraft noise levels during the night and used the L_night_ indicator defined as the weighted average of sound levels during the night (22:00 to 6:00 or 23:00 to 7:00) [51].

### Annoyance due to aircraft noise

ISO/ICBEN (International Commission on the Biological Effects of Noise) recommends using the following question in community studies to assess aircraft noise annoyance [52]: “Thinking about the last 12 months, when you are here at home, how much does aircraft noise bother, disturb or annoy you?”. HYENA and DEBATS both followed this recommandation.

HYENA then used the standard numeric scale for night and daytime annoyance separately (range 0–10). The participants included in the present study were considered to be highly annoyed when their average score between night and day was ≥8. In the sensitivity analyses, they were considered highly annoyed when their highest score between night and day was ≥8.

DEBATS used the standard verbal scale with five possible responses, namely extremely, very, moderately, slightly or not at all. The participants included in the present study were considered highly annoyed when they answered extremely or very.

The standardized definition of “highly annoyed people”, using either the numeric or verbal scale, has been recommended by ICBEN to make the scales comparable and has been adopted by a large majority of studies dealing with noise annoyance. Therefore, in this study, the dichotomization and harmonization between the two annoyance scales was carried out in accordance with these recommendations [53].

### Noise sensitivity

HYENA used the short-form of the Weinstein scale in 10 items to assess noise sensitivity [54]. Participants were expected to indicate their degree of agreement (from 1 to 6) with different noise statements and were then assigned an overall score based on the 10 items.

DEBATS used the following five-point question to assess noise sensitivity: “Regarding noise in general, compared to people around you, do you think that you are: much less sensitive than, less sensitive than, as sensitive as, a little more sensitive than or more sensitive than people around you?”

Then a common variable was derived for the pooled analyses. The score corresponding to the only direct item on noise sensitivity in HYENA was matched to the different response modalities in DEBATS in order to create a variable with three categories: “low noise sensitive” which includes HYENA scores 1 and 2 as well as the categories “much less sensitive” and “less sensitive” in DEBATS; “medium noise sensitive” which includes HYENA scores 3 and 4 and the category “as sensitive” in DEBATS; and “high noise sensitive” which includes HYENA scores 5 and 6 together with the categories “a little more sensitive” and “ much more sensitive” in DEBATS.

### Medication use

During the interview, participants were invited to report all prescribed and non-prescribed medications used in the last 2 weeks (HYENA) and the last 12 months (DEBATS) prior to the interview. Each medication was coded according to the Anatomical Therapeutic Chemical Classification System (ATC) [55] proposed by the World Health Organization (WHO). Based on this classification, seven dichotomized variables corresponding to the following groups of medications were defined and analysed separately:

- Antihypertensives (ATC codes C02A, C02C, C02D, C02N, C03A, C03B, C03C, C03E, C07, C08, C09A, C09B, C09C, C09D);
- Antacids (ATC codes A02);
- Anxiolytics (ATC codes N05B);
- Hypnotic and sedatives drugs (ATC codes N05C);
- Anxiolytics, and hypnotic and sedative drugs (ATC codes N05B, N05C). Anxiolytics, and hypnotics and sedatives were combined into one group because anxiolytics can be prescribed in the short term at higher doses to produce hypnotic effects;
- Antidepressants (ATC codes N06A);
- Antasthmatics (ATC codes R03).

### Statistical analysis

Logistic regression models were used with medication groups as outcome variables, and aircraft noise levels (M0 model), aircraft noise annoyance (M1 model), or noise sensitivity (M2 model) as exposure variables, and potential confounders as covariates.

The main potential confounding factors were obtained during the face-to-face interview and introduced a priori in the models: gender, age, body mass index (BMI), alcohol consumption, smoking habits, physical activity, education level, and country. In addition, an interaction term between country and each of the three factors of interest (aircraft noise levels, aircraft noise annoyance and noise sensitivity) was included in M0, M1 and M2 models in order to take into account cultural differences that may moderate noise exposure, noise annoyance or noise sensitivity [56]. Models where the interaction was not statistically significant were not presented.

The mediating or modifying role of aircraft noise annoyance and noise sensitivity has been studied according to the recommendations of Baron and Kenny [57]. Model results including aircraft noise levels (M0 model), aircraft noise annoyance (M1 model), or noise sensitivity (M2 model), and aircraft noise levels and aircraft noise annoyance (M3 model) or aircraft noise levels and noise sensitivity (M4 model), as well as potential confounders as covariates, were compared to evaluate a possible mediating effect of aircraft noise annoyance or of noise sensitivity. To assess the possible modifying effects of aircraft noise annoyance or of noise sensitivity, an interaction term between aircraft noise levels at night (L_night_) and aircraft noise annoyance (M5 model), and between aircraft noise exposure at night (L_night_) and noise sensitivity (M6 model) was included in the M0 model.

Sensitivity analyses were performed with another common variable in three categories for sensitivity to noise: “low noise sensitive” which includes the first tertile of the country-standardized mean of the overall Weinstein scale score used for HYENA participants as well as the “much less sensitive” and “less sensitive” categories in DEBATS; “medium noise sensitive” which includes the second above-mentioned tertile for HYENA participants and the category “as sensitive” in DEBATS; and “high noise sensitive” which includes the third tertile for HYENA participants together with the categories “a little more sensitive” and “ much more sensitive” in DEBATS.

The logistic procedure of SAS software V. 9.4 (SAS Institute, Cary NC) was used for all statistical analyses. Adjusted odds-ratios (ORs) and their 95% confidence intervals (CIs) are reported.

## Results

**Fig. 1:**
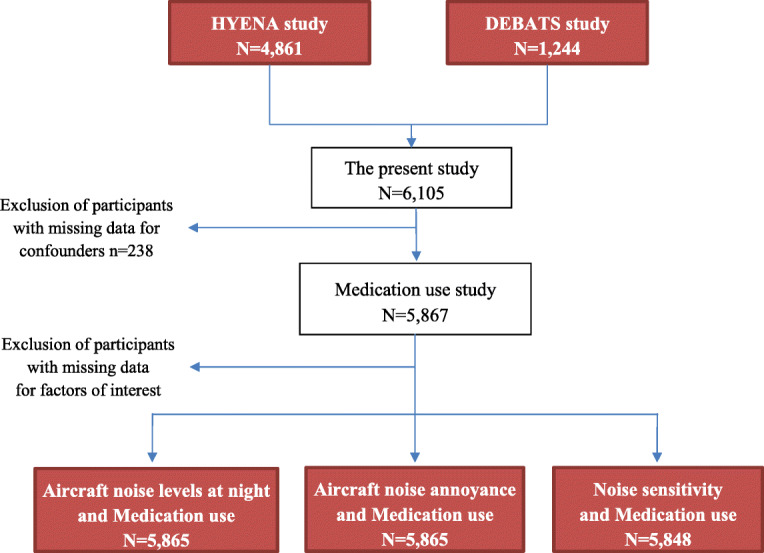
Study population

Statistical analyses covered 5867 participants, with completed information for all the confounding factors included in the models (Fig. 1). Participation rates varied by country, ranging from approximately 30% in France, Germany, Italy, and the UK to 46% in the Netherlands, 56% in Greece, and 78% in Sweden.

Overall, 25% of the participants used antihypertensive medication: this varied from 16% in France to 33% in Germany. Prevalence for the other medication groups ranged from 3 to 9%, with significant differences between countries. Almost 20% of participants reported being highly annoyed by aircraft noise, from 10% in Sweden to 43% in Greece. Approximately 35% of the participants reported low sensitivity to noise, 32% medium sensitivity and 33% high sensitivity. Participants from Sweden were the least sensitive to noise, while Italian participants were the most sensitive to noise (Table 1).

**Table 1:** Prevalence of medication use and factors of interest by country

	UK N* = 585 (288/297)	GE N* = 969 (502/467)	NL N* = 871 (445/426)	SW N* = 991 (481/510)	GR N* = 618 (337/281)	IT N* = 612 (284/328)	FR N* = 1221 (683/538)	Overall N* = 5867 (3020/2847)	p
**Antihypertensive medication** ^a^	181 *(30.9)*	315 *(32.5)*	199 *(22.8)*	221 *(22.3)*	188 *(30.4)*	180 *(29.4)*	195 *(16.0)*	1479 *(25.2)*	**< 0.01**
**Antacids** ^a^	62 *(10.6)*	78 *(8.0)*	83 *(9.5)*	38 *(3.8)*	29 *(4.7)*	35 *(5.7)*	68 *(5.6)*	393 *(6.7)*	**< 0.01**
**Anxiolytics** ^a^	6 *(1.0)*	5 *(0.5)*	42 *(4.8)*	11 *(1.1)*	26 *(4.2)*	55 *(9.0)*	75 *(6.1)*	220 *(3.8)*	**< 0.01**
**Hypnotics** ^a^	13 *(2.2)*	10 *(1.0)*	31 *(3.6)*	30 *(3.0)*	1 *(0.2)*	12 *(2.0)*	48 *(3.9)*	145 *(2.5)*	**< 0.01**
**Anxiolytics, hypnotics and sedatives** ^a^	19 *(3.2)*	14 *(1.4)*	70 *(8.0)*	37 *(3.7)*	27 *(4.4)*	64 *(10.5)*	114 *(9.3)*	345 *(5.9)*	**< 0.01**
**Antidepressants** ^a^	38 *(6.5)*	29 *(3.0)*	48 *(5.5)*	53 *(5.3)*	7 *(1.1)*	18 *(2.9)*	44 *(3.6)*	237 *(4.0)*	**< 0.01**
**Antasthmatics** ^a^	50 *(8.5)*	43 *(4.4)*	44 *(5.1)*	50 *(5.0)*	19 *(3.1)*	10 *(1.6)*	42 *(3.4)*	258 *(4.4)*	**< 0.01**
**Aircraft noise levels (L**_**night**_**)** ^b^	49.3 *(10.5)*	40.2 *(10.0)*	42.2 *(8.9)*	39.5 *(7.9)*	41.7 *(4.6)*	35.4 *(6.4)*	45.1 *(6.4)*	42.0 *(8.9)*	**< 0.01**
**Aircraft noise annoyance** ^a^									**< 0.01**
*Not highly annoyed*	394 *(67.4)*	816 *(84.2)*	774 *(88.9)*	893 *(90.1)*	349 *(56.7)*	475 *(77.6)*	999 *(81.8)*	4700 *(80.1)*	
*Highly annoyed*	191 *(32.6)*	153 *(15.8)*	97 *(11.1)*	98 *(9.9)*	267 *(43.3)*	137 *(22.4)*	222 *(18.2)*	1165 *(19.9)*	
**Noise sensitivity** ^a^									**< 0.01**
*Low*	132 *(22.8)*	389 *(40.2)*	360 *(41.4)*	516 *(52.1)*	209 *(33.9)*	199 *(32.6)*	270 *(22.3)*	2075 *(35.5)*	
*Medium*	192 *(33.2)*	282 *(29.1)*	290 *(33.3)*	222 *(22.4)*	162 *(26.3)*	130 *(21.3)*	582 *(48.0)*	1860 *(31.8)*	
*High*	255 *(44.0)*	297 *(30.7)*	220 *(25.3)*	253 *(25.5)*	245 *(39.8)*	282 *(46.2)*	361 *(29.8)*	1913 *(32.7)*	

(*UK* United Kingdom; *GE* Germany; *NL* The Netherlands; *SW* Sweden; *GR* Greece; *IT* Italy; *FR* France)

*Number of participants (men/women)

^a^ N (%)

^b^ mean (± SD)

The distribution of the participants was significantly different according to aircraft noise levels for antacid use, age, education, physical activity, alcohol consumption and smoking habits. The distribution of the participants was also different between highly annoyed and not highly annoyed participants for the use of antihypertensive/anxiolytic/anxiolytic, hypnotic and sedative/antasthmatic medication, age, education level, alcohol consumption and smoking habits. Finally, the distribution of the participants differed by noise sensitivity categories for almost all medication use (except antacids and antasthmatics), gender, age, education, BMI, physical activity, alcohol consumption, and smoking habits (Table 2).

**Table 2:** Study population characteristics by categories of aircraft noise levels, aircraft noise annoyance and noise sensitivity

	Aircraft noise levels in dB(A) (L_**night**_)					Aircraft noise annoyance			Noise sensitivity			
	< 40	40–44	45–49	≥50	p	Not highly annoyed	Highly annoyed	p	Low sensitivity	Medium sensitivity	High sensitivity	p
**Antihypertensive medication**					0.39			**< 0.01**				**< 0.01**
*No*	1926 *(43.9)*	672 *(15.3)*	795 *(18.1)*	995 *(22.7)*		3578 *(81.5)*	810 *(18.5)*		1563 *(35.7)*	1428 *(32.6)*	1384 *(31.6)*	
*Yes*	633 *(42.8)*	214 *(14.5)*	265 *(17.9)*	367 *(24.8)*		1122 *(76.0)*	355 *(24.0)*		512 *(34.8)*	432 *(29.3)*	529 *(35.9)*	
**Antacids**					**0.01**			0.12				0.17
*No*	2395 *(43.8)*	834 *(15.2)*	1000 *(18.3)*	1245 *(22.7)*		4397 *(80.4)*	1075 *(19.6)*		1948 *(35.7)*	1742 *(31.9)*	1769 *(32.4)*	
*Yes*	164 *(41.7)*	52 *(13.2)*	60 *(15.3)*	117 *(29.8)*		303 *(77.1)*	90 *(22.9)*		127 *(32.6)*	118 *(30.3)*	144 *(37.0)*	
**Anxiolytics**					0.12			**< 0.01**				**< 0.01**
*No*	2476 *(43.8)*	842 *(14.9)*	1017 *(18.0)*	1312 *(23.2)*		4542 *(80.5)*	1103 *(19.5)*		2031 *(36.1)*	1785 *(31.7)*	1813 *(32.2)*	
*Yes*	83 *(37.7)*	44 *(20.0)*	43 *(19.5)*	50 *(22.7)*		158 *(71.8)*	62 *(28.2)*		44 *(20.1)*	75 *(34.2)*	100 *(45.7)*	
**Hypnotics**					0.21			0.13				**< 0.01**
*No*	2491 *(43.5)*	871 *(15.2)*	1038 *(18.1)*	1322 *(23.1)*		4591 *(80.3)*	1129 *(19.7)*		2047 *(35.9)*	1814 *(31.8)*	1842 *(32.3)*	
*Yes*	68 *(46.9)*	15 *(10.3)*	22 *(15.2)*	40 *(27.6)*		109 *(75.2)*	36 *(24.8)*		28 *(19.3)*	46 *(31.7)*	71 *(49.0)*	
**Anxiolytics, hypnotics and sedatives**					0.59			**< 0.01**				**< 0.01**
*No*	2418 *(43.8)*	828 *(15.0)*	1000 *(18.1)*	1276 *(23.1)*		4448 *(80.6)*	1072 *(19.4)*		2007 *(36.5)*	1747 *(31.7)*	1750 *(31.8)*	
*Yes*	141 *(40.9)*	58 *(16.8)*	60 *(17.4)*	86 *(24.9)*		252 *(73.0)*	93 *(27.0)*		68 *(19.8)*	113 *(32.8)*	163 *(47.4)*	
**Antidepressants**					0.14			0.61				**< 0.01**
*No*	2445 *(43.4)*	862 *(15.3)*	1019 *(18.1)*	1304 *(23.2)*		4507 *(80.1)*	1121 *(19.9)*		2025 *(36.1)*	1788 *(31.9)*	1798 *(32.0)*	
*Yes*	114 *(48.1)*	24 *(10.1)*	41 *(17.3)*	58 *(24.5)*		193 *(81.4)*	44 *(18.6)*		50 *(21.1)*	72 *(30.4)*	115 *(48.5)*	
**Antasthmatics**					0.18			**0.03**				0.45
*No*	2449 *(43.7)*	855 *(15.2)*	1016 *(18.1)*	1289 *(23.0)*		4507 *(80.4)*	1100 *(19.6)*		1974 *(35.3)*	1782 *(31.9)*	1834 *(32.8)*	
*Yes*	110 *(42.6)*	31 *(12.0)*	44 *(17.1)*	73 *(28.3)*		193 *(74.8)*	65 *(25.2)*		101 *(39.1)*	78 *(30.2)*	79 *(30.6)*	
**Gender** ^**a**^					0.26			0.96				**< 0.01**
*Men*	1274 *(44.7)*	415 *(14.6)*	518 *(18.2)*	640 *(22.5)*		2280 *(80.1)*	566 *(19.9)*		1137 *(40.1)*	905 *(31.9)*	792 *(27.9)*	
*Women*	1285 *(42.5)*	471 *(15.6)*	542 *(17.9)*	722 *(23.9)*		2420 *(80.2)*	599 *(19.8)*		938 *(31.1)*	955 *(31.7)*	1121 *(37.2)*	
**Age** ^**b**^	56.8 *(8.6)*	55.6 *(11.1)*	56.2 *(10.4)*	55.7 *(10.9)*	**< 0.01**	56.1 *(10.1)*	57.0 *(9.1)*	**< 0.01**	56.7 *(9.5)*	55.7 *(10.8)*	56.3 *(9.5)*	**0.01**
**Education** ^**a,c**^					**< 0.01**			**< 0.01**				**< 0.01**
*1st qrt*	550 *(35.0)*	329 *(20.9)*	308 *(19.6)*	386 *(24.5)*		1216 *(77.4)*	355 *(22.6)*		577 *(36.9)*	513 *(32.8)*	475 *(30.4)*	
*2nd qrt*	584 *(41.7)*	143 *(10.2)*	260 *(18.5)*	415 *(29.6)*		1171 *(83.5)*	231 *(16.5)*		543 *(38.9)*	446 *(31.9)*	408 *(29.2)*	
*3rd qrt*	668 *(48.5)*	165 *(12.0)*	229 *(16.6)*	316 *(22.9)*		1118 *(81.1)*	260 *(18.9)*		480 *(34.9)*	416 *(30.2)*	480 *(34.9)*	
*4rd qrt*	757 *(50.0)*	249 *(16.4)*	263 *(17.4)*	245 *(16.2)*		1195 *(78.9)*	319 *(21.1)*		475 *(31.5)*	485 *(32.1)*	550 *(36.4)*	
**BMI** ^**b**^	26.9 *(4.7)*	26.8 *(5.0)*	26.9 *(4.6)*	27.2 *(4.8)*	0.07	26.9 *(4.8)*	27.2 *(4.6)*	0.06	27.3 *(4.8)*	26.7 *(4.6)*	26.8 *(4.7)*	**< 0.01**
**Physical activity** ^**a**^					**< 0.01**			0.21				0.45
*No or little*	1244 *(40.8)*	461 *(15.1)*	568 *(18.6)*	773 *(25.4)*		2421 *(79.5)*	624 *(20.5)*		1063 *(35.0)*	989 *(32.5)*	988 *(32.5)*	
*Regular*	1315 *(46.6)*	425 *(15.1)*	492 *(17.4)*	589 *(20.9)*		2279 *(80.8)*	541 *(19.2)*		1012 *(36.0)*	871 *(31.0)*	925 *(32.9)*	
**Alcohol (units/week)** ^**a**^					**< 0.01**			**< 0.01**				**< 0.01**
*teetotaller*	645 *(38.6)*	263 *(15.8)*	324 *(19.4)*	437 *(26.2)*		1288 *(77.2)*	380 *(22.8)*		548 *(33.0)*	509 *(30.6)*	*606 (36.4)*	
*1–7*	1248 *(44.8)*	411 *(14.8)*	512 *(18.4)*	614 *(22.0)*		2246 *(80.6)*	539 *(19.4)*		996 *(35.8)*	895 *(32.2)*	889 *(32.0)*	
*8–14*	396 *(47.6)*	118 *(14.2)*	140 *(16.8)*	178 *(21.4)*		705 *(84.7)*	127 *(15.3)*		322 *(38.9)*	282 *(34.1)*	224 *(27.1)*	
*> 14*	270 *(46.5)*	94 *(16.2)*	84 *(14.5)*	133 *(22.9)*		461 *(79.5)*	119 *(20.5)*		209 *(36.2)*	174 *(30.2)*	194 *(33.6)*	
**Smoking habits (units/day)** ^**a**^					**< 0.01**			**< 0.01**				**< 0.01**
*non-smoker*	1028 *(40.2)*	380 *(42.9)*	470 *(44.3)*	625 *(45.9)*		1998 *(42.5)*	504 *(43.3)*		764 *(36.8)*	836 *(44.9)*	894 *(46.7)*	
*exsmoker*	943 *(36.9)*	260 *(29.3)*	321 *(30.3)*	415 *(30.5)*		1587 *(33.8)*	351 *(30.1)*		717 *(34.6)*	606 *(32.6)*	611 *(31.9)*	
*0–10*	206 *(8.1)*	85 *(9.6)*	95 *(9.0)*	143 *(10.5)*		414 *(8.8)*	115 *(9.9)*		202 *(9.7)*	171 *(9.2)*	152 *(7.9)*	
*11–20*	235 *(9.2)*	85 *(9.6)*	108 *(10.2)*	118 *(8.7)*		435 *(9.3)*	111 *(9.5)*		238 *(11.5)*	138 *(7.4)*	169 *(8.8)*	
*> 20*	147 *(5.7)*	76 *(8.6)*	66 *(6.2)*	61 *(4.5)*		266 *(5.7)*	84 *(7.2)*		154 *(7.4)*	109 *(5.9)*	87 *(4.5)*	
**Aircraft noise annoyance**					**< 0.01**							**< 0.01**
*Not highly annoyed*	2327 *(49.5)*	643 *(13.7)*	780 *(16.6)*	950 *(20.2)*					1853 *(39.6)*	1478 *(31.6)*	1353 *(28.9)*	
*Highly annoyed*	231 *(19.8)*	242 *(20.8)*	280 *(24.0)*	412 *(35.4)*					220 *(18.9)*	382 *(32.9)*	560 *(48.2)*	
**Noise sensitivity**					**< 0.01**			**< 0.01**				
*Low*	983 *(47.4)*	250 *(12.0)*	392 *(18.9)*	450 *(21.7)*		1853 *(89.4)*	220 *(10.6)*					
*Medium*	729 *(39.2)*	337 *(18.1)*	320 *(17.2)*	474 *(25.5)*		1478 *(79.5)*	382 *(20.5)*					
*High*	840 *(43.9)*	294 *(15.4)*	347 *(18.1)*	432 *(22.6)*		1353 *(70.7)*	560 *(29.3)*					
**TOTAL**	**2559** *(43.6)*	**886** *(15.1)*	**1060** *(18.1)*	**1362** *(23.2)*		**4700** *(80.1)*	**1165** *(19.9)*		**2075** *(35.5)*	**1860** *(31.8)*	**1913** *(32.7)*	

^a^ N *(%)*

^b^ mean (± SD)

^c^ coded in quartiles of the number of years of education previously standardized by country means

**Fig. 2:**
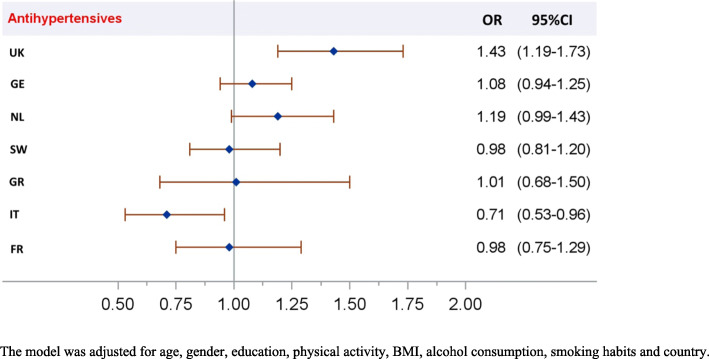
Odds ratios (OR) for the use of antihypertensive medication in relation to aircraft noise levels at night (M0 model including the interaction between aircraft noise levels and country)

A 10 dB(A)-increase in night-time exposure to aircraft noise was significantly associated with increased use of antihypertensive medication (OR = 1.10, 95%CI 1.01–1.18) (Table 3, M0 model). However, a significant interaction was found for antihypertensive medication use between aircraft noise levels at night (L_night_) and country (Fig. 2, M0 model): the association was positively significant in the UK (OR = 1.43, 95%CI 1.19–1.73 for a 10 dB(A)-increase in L_night_), and negatively significant in Italy (OR = 0.71, 95%CI 0.53–0.96 for a 10 dB(A)-increase in L_night_). No association was found with other medications.

**Table 3:** Odds ratios for medication use in relation to a 10 dB(A)-increase in aircraft noise exposure at night (L_night_) and/or aircraft noise annoyance or noise sensitivity

	Antihypertensive medication		Antacids		Anxiolytics		Hypnotics		Anxiolytics, hypnotics and sedatives		Antidepressants		Antasthmatics	
	*OR*	*95%CI*	*OR*	*95%CI*	*OR*	*95%CI*	*OR*	*95%CI*	*OR*	*95%CI*	*OR*	*95%CI*	*OR*	*95%CI*
**M0 model**														
**L**_**night**_	**1.10**	**(1.01–1.18)**	1.02	(0.90–1.16)	1.15	(0.94–1.39)	0.88	(0.71–1.09)	1.03	(0.89–1.20)	0.92	(0.79–1.08)	1.02	(0.88–1.18)
**M1 model**														
**Aircraft noise annoyance**														
*Highly* vs *not highly annoyed*	**1.33**	**(1.14–1.56)**	1.20	(0.93–1.55)	**1.48**	**(1.08–2.05)**	**1.60**	**(1.07–2.39)**	**1.56**	**(1.20–2.03)**	1.02	(0.72–1.44)	**1.44**	**(1.07–1.96)**
**M2 model**														
**Noise sensitivity**														
*Medium* vs *low*	1.06	(0.90–1.25)	1.03	(0.79–1.35)	**1.66**	**(1.12–2.47)**	**1.78**	**(1.09–2.92)**	**1.70**	**(1.24–2.35)**	**1.84**	**(1.26–2.68)**	0.86	(0.63–1.17)
*High* vs *low*	**1.30**	**(1.11–1.52)**	**1.32**	**(1.02–1.71)**	**2.32**	**(1.59–3.38)**	**3.37**	**(2.13–5.33)**	**2.78**	**(2.05–3.77)**	**2.99**	**(2.10–4.25)**	0.85	(0.62–1.16)
**M3 model**														
**L**_**night**_	1.06	(0.98–1.15)	1.00	(0.88–1.13)	0.97	(0.83–1.13)	1.09	(0.88–1.33)	0.82	(0.66–1.02)	0.92	(0.78–1.08)	0.97	(0.83–1.13)
**Aircraft noise annoyance**														
*Highly* vs *not highly annoyed*	**1.29**	**(1.10–1.53)**	1.20	(0.92–1.57)	**1.43**	**(1.03–2.00)**	**1.80**	**(1.18–2.72)**	**1.59**	**(1.21–2.09)**	1.05	(0.73–1.51)	**1.47**	**(1.07–2.02)**
**M4 model**														
**L**_**night**_	**1.09**	**(1.01–1.18)**	1.02	(0.90–1.15)	1.02	(0.88–1.19)	1.13	(0.92–1.37)	0.88	(0.71–1.09)	0.92	(0.78–1.08)	1.02	(0.88–1.18)
**Noise sensitivity**														
*Medium* vs *low*	1.06	(0.90–1.25)	1.03	(0.79–1.35)	**1.66**	**(1.12–2.46)**	**1.78**	**(1.09–2.92)**	**1.70**	**(1.24–2.35)**	**1.84**	**(1.26–2.69)**	0.86	(0.63–1.18)
*High* vs *low*	**1.30**	**(1.11–1.52)**	**1.32**	**(1.02–1.71)**	**2.31**	**(1.59–3.37)**	**3.36**	**(2.13–5.31)**	**2.78**	**(2.05–3.77)**	**2.97**	**(2.08–4.22)**	0.85	(0.62–1.16)

**M0 model** = L_night_ + confounders; **M1 model** = aircraft noise annoyance + confounders; **M2 model** = noise sensitivity + confounders; **M3 model** = L_night_ + aircraft noise annoyance + confounders; **M4 model** = L_night_ + noise sensitivity + confounders

For each model, confounders were gender, age, education, physical activity, BMI, alcohol consumption, smoking habits and country

Aircraft noise annoyance was significantly associated with the use of antihypertensive medication (OR = 1.33, 95%CI 1.14–1.56), anxiolytics (OR = 1.48, 95%CI 1.08–2.05), hypnotics and sedatives (OR = 1.60, 95%CI 1.07–2.39) and antasthmatics (OR = 1.44, 95%CI 1.07–1.96) (Table 3, M1 model), with no difference between countries.

**Fig. 3:**
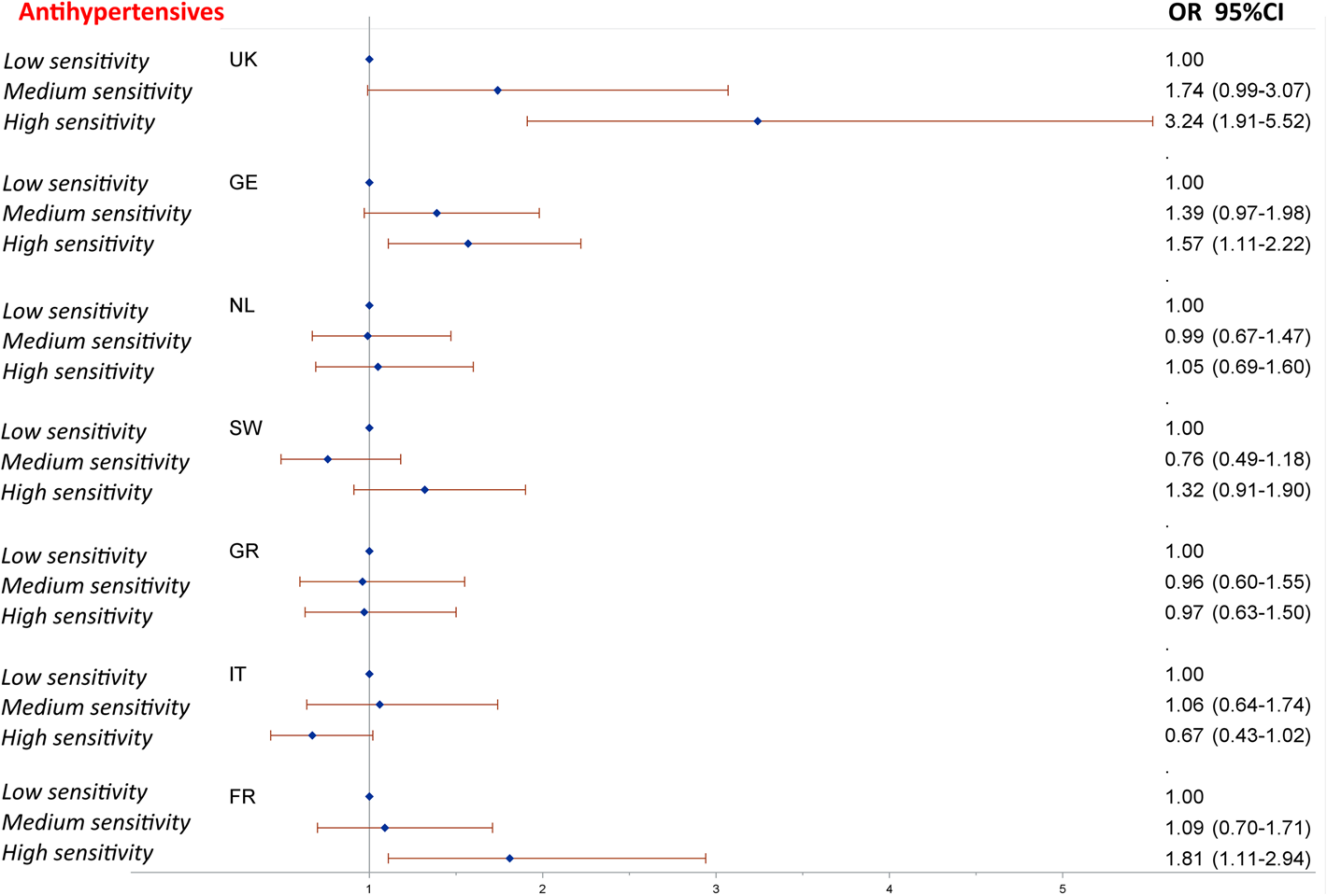
Odds ratios (OR) for the use of antihypertensive medication in relation to noise sensitivity (M2 model including the interaction between noise sensitivity and country)

Noise sensitivity was significantly associated with almost all medications, with the exception of the use of antasthmatics, showing an increase in ORs with the level of noise sensitivity (Table 3, M2 model). There were, however, differences between countries in the use of antihypertensive medication, as shown by the significant interaction between noise sensitivity and country. ORs were non-significant and close to 1 for the Netherlands, Sweden, Greece and Italy, but significantly greater than 1 for Germany, France and the UK, where the association was the strongest (OR = 1.74, 95%CI 0.99–3.07, and OR = 3.24, 95%CI 1.91–5.52 for medium and high sensitivity respectively, both compared to low sensitivity) (Fig. 3, M2 model).

The OR between aircraft noise levels and antihypertensive medication became slightly lower and non-significant in the M3 model including aircraft noise annoyance (Table 3, M3 model), compared to the M0 model (Table 3, M0 model). It remained similar in the M4 model including noise sensitivity (Table 3, M4 model) and in the M0 model (Table 3, M0 model).

The OR between aircraft noise levels and antihypertensive medication was significantly higher in the highly annoyed participants (OR = 1.27, 95%CI 1.06–1.53 for a 10-dB(A) increase in L_night_) than in those who were not highly annoyed (OR = 1.03, 95%CI 0.94–1.12) (p of the interaction term = 0.03). Although this interaction was not significant for other medications, the same trend was observed with higher ORs for a 10-dB(A) increase in L_night_ in the highly annoyed participants compared to those who were not highly annoyed (Table 4, M5 model).

**Table 4:** Odds ratios (OR) for medication use in relation to a 10 dB(A)-increase in aircraft noise exposure at night (L_night_) after taking into account the interaction between L_night_ and aircraft noise annoyance and between L_night_ and noise sensitivity

	N	Antihypertensive medication		Antacids		Anxiolytics		Hypnotics		Anxiolytics, hypnotics and sedatives		Antidepressants		Antasthmatics	
		OR	*95%CI*	OR	*95%CI*	OR	*95%CI*	OR	*95%CI*	OR	*95%CI*	OR	*95%CI*	OR	*95%CI*
**Aircraft noise levels at night (L**_**night**_**)**^**a**^															
For not highly annoyed people	**4700**	1.03	(0.94–1.12)	0.98	(0.85–1.12)	1.05	(0.84–1.31)	**0.77**	**(0.60–0.99)**	0.93	(0.78–1.10)	0.93	(0.78–1.11)	0.93	(0.79–1.10)
For highly annoyed people	**1165**	**1.27**	**(1.06–1.53)**	1.11	(0.84–1.49)	1.24	(0.83–1.86)	1.02	(0.64–1.61)	1.14	(0.84–1.57)	0.84	(0.57–1.24)	1.14	(0.82–1.60)
*p*_*interaction*_		***0.03***		*0.40*		*0.46*		*0.29*		*0.23*		*0.63*		*0.27*	
**Aircraft noise levels at night (L**_**night**_**)**^**b**^															
For low noise sensitivity	**2075**	0.94	(0.83–1.06)	0.99	(0.82–1.21)	**1.47**	**(1.02–2.11)**	0.74	(0.48–1.16)	1.08	(0.81–1.44)	1.10	(0.81–1.49)	0.86	(0.69–1.08)
For medium noise sensitivity	**1860**	1.03	(0.90–1.18)	0.98	(0.79–1.21)	1.09	(0.80–1.49)	0.83	(0.58–1.20)	0.96	(0.75–1.24)	1.00	(0.77–1.31)	1.16	(0.90–1.50)
For high noise sensitivity	**1913**	**1.37**	**(1.21–1.56)**	1.08	(0.89–1.31)	0.99	(0.75–1.32)	1.01	(0.75–1.35)	1.04	(0.84–1.29)	0.80	(0.64–1.00)	1.11	(0.86–1.42)
*p*_*interaction*_		***< 0.01***		*0.76*		*0.21*		*0.47*		*0.81*		*0.19*		*0.15*	

^a^ M5 model: M0 model including the interaction between L_night_ and noise annoyance

^b^ M6 model: M0 model including the interaction between L_night_ and noise sensitivity

Both models were adjusted for age, gender, education, physical activity, BMI, alcohol consumption, smoking habits and country

The association between aircraft noise levels at night and the use of antihypertensive medication was also found to increase with the noise sensitivity level of the participants (OR = 0.94, 95%CI 0.83–1.06; OR = 1.03, 95%CI 0.90–1.18; OR = 1.37, 95%CI 1.21–1.56, with a 10-dB(A) increase in L_night_ in individuals with low, medium, and high sensitivity respectively; p of the interaction term < 0.01) (Table 4, M6 model).

Similar results were obtained using Weinstein tertiles for HYENA participants to define the common noise sensitivity variable.

## Discussion

Combining HYENA and DEBATS datasets allowed this study to obtain such a large population, and therefore a higher statistical power than any other study, to evaluate the extent to which the association between aircraft noise levels and medication use can be mediated and/or modified by aircraft noise annoyance or noise sensitivity.

Although only a few studies in the literature addressed this issue, their results are similar to those in this study. Associations between aircraft noise exposure and medication use have been found between aircraft noise levels and the use of antihypertensive or cardiac medication, hypnotics and sedatives, or anxiolytics [17–20], but not with psychotropic medication [33]. In the HYENA study, Floud et al. showed a significant association between aircraft noise levels and the use of antihypertensive medication in the UK only (OR = 1.34, 95%CI 1.14–1.57 for a 10-dB(A) increase in L_night_), as well as an association between aircraft noise levels and the use of anxiolytics, without any differences between countries [17]. The present pooled analysis of the HYENA and DEBATS datasets confirmed the association between aircraft noise levels at night and the use of antihypertensive medication in the UK only (OR = 1.43, 95%CI 1.19–1.73 for a 10-dB(A) increase in L_night_), but failed to report an association for the use of anxiolytics. However, Floud et al. used a multilevel logistic regression model with a hierarchical structure to model possible differences between countries in prescribing, whereas we used a logistic regression model including country as a confounding factor and then including an interaction term between country and factors of interest. In the present study, multilevel logistic regression models were also fitted and their Akaike information criterions (AICs) were compared. For all medications, the comparison of AICs led to prefer classical logistic regression models including country as a confounding factor or an interaction term between country and factors of interest. Moreover, the estimates of ORs and *p*-values were very close for multilevel and classical logistic regression models.

When a significant association was found between aircraft noise annoyance and several medication uses (antihypertensives, anxiolytics, hypnotics, antasthmatics), the results are partly consistent with those of the few studies carried out on the subject. Watkins et al. found that the use of non-prescribed medication and the use of psychotropic drugs was significantly higher among highly annoyed participants than among less annoyed participants near London-Heathrow airport [33]. Significant prevalence risk ratios have been reported for the use of antihypertensive medication in relation to aircraft noise annoyance around Stockholm Arlanda Airport [58]. Floud et al. found, in the HYENA study, significant associations between aircraft noise annoyance and the use of antihypertensive medication, anxiolytics, and anxiolytics combined with hypnotics [17]. While to date there is little evidence of a direct association between noise exposure and asthma [59–61], in contrast to Floud et al., a significant association was observed between aircraft noise annoyance and the use of anti-asthmatics. However, this latter study adjusted aircraft noise levels for road levels, whereas we did not because these two noise levels were highly correlated.

The present results are also partly consistent with those of the few previous studies showing an increased gradient in the use of antihypertensive, anxiolytic-hypnotic-sedative and antidepressant medications with higher levels of noise sensitivity. However, the various studies were conducted in different contexts, with different settings and included participants exposed to different noise sources (not just aircraft noise). Clearly, this may affect the associations between noise sensitivity and health outcomes. The gradient in the use of antihypertensive medication did not hold for all countries but only for the UK, Germany and France. In the literature, increased noise sensitivity has been associated with increased blood pressure [39], health complaints (including cardiac complaints) [31, 40], hypertension [42], psychological distress [40] and the use of psychotropic drugs (sleeping pills, tranquilizers and pain relievers) [43]. The gradient observed for the use of antihypertensive medication with higher levels of noise sensitivity in the UK echoes the association found between aircraft noise levels and the use of antihypertensive medication in the pooled analyses of the HYENA and DEBATS studies [38]. Noise sensitivity has been shown to be related to psychological disorders [40] - this being relevant to the association with medication.

When both aircraft noise annoyance and aircraft noise levels at night (L_night_) were included in the M0 model (M4 model), the associations between aircraft noise levels and the use of antihypertensives became lower and non-significant. This suggests a possible mediating effect of aircraft noise annoyance in the relationship between aircraft noise levels and the use of antihypertensives. Aircraft noise annoyance has been previously reported as a possible mediator or intermediate factor between aircraft noise exposure and mental health, but has never been reported for other health outcomes or physiological factors [27, 34, 35].

When both noise sensitivity and aircraft noise levels at night (L_night_) were included in the M0 model (M5 model), the results remained very similar, suggesting that noise sensitivity cannot be considered as a mediator in the relationship between noise levels and medication use. However, more studies are needed to conclude as there are no other studies in the literature on the role for noise sensitivity.

This study found significant interactions between aircraft noise annoyance and aircraft noise at night (L_night_), and between noise sensitivity and aircraft noise at night (L_night_) for the use of antihypertensives. This suggests a modifying effect of aircraft noise annoyance and noise sensitivity in the relationship between aircraft noise levels and the use of antihypertensive medication. Aircraft noise annoyance has already been shown to modify the relationship between aircraft noise levels and the risk of hypertension [36–38], while noise sensitivity has already been found to modify the associations between aircraft noise levels and somatic symptoms [44], general health [34], and psychological disorders [45].

The low response rate in most of the countries participating in the present study could be a possible source of selection bias. However, non-response to the survey was random, with only minor differences between the demographic and socioeconomic characteristics of respondents and non-respondents [5, 7].

Some of the associations observed in this study between aircraft noise annoyance and medication use or between noise sensitivity and medication use could be the result of reporting bias. Indeed participants taking medication may over-report noise annoyance or noise sensitivity because they attribute their health problems to external factors [62]. Aircraft noise annoyance has been found to be associated with the use of antasthmatic medication, and noise sensitivity has been found to be associated with the use of antidepressants, while no association was found with noise levels for these medications. It is possible that participants, who are in poor health and being treated for health problems, may be more vulnerable to environmental stressors or may be unable to leave their homes to avoid noise exposure, which could result in higher noise annoyance or noise sensitivity [33].

Recall bias may also occur due to the difference in recall time for previous medications between the two studies: 2 weeks in the HYENA study vs. 12 months in the DEBATS study. Nevertheless, when participants are asked to report medications they have taken in the past 12 months, it can be assumed that due to recall bias, they respond for a shorter and more recent period. Furthermore, this difference in recall would not affect chronic conditions, which require regular use of medications such as hypertension. It would however affect more occasional use of medication and conditions, which are presumably more prone to forgetfulness. Although low prevalence for some ATC-groups medication has been reported in some countries, resulting in a lack of statistical power in statistical analyses, pooled analyses can address this problem, while taking into account this difference in collection.

The prevalence of medication use was different in the participating countries (Table 1). Prescribing and access to medicines are known to differ between European countries, both in terms of quantity and category of medicines [63], and in terms of co-payment due to differences between European health systems [64]. To account for differences in prevalence between countries, we used models including the country as a confounding factor and then including a term for the interaction between country and aircraft noise annoyance or between country and noise sensitivity. This interaction was significant when investigating aircraft noise levels at night or noise sensitivity in relation to the use of antihypertensive medication only. The difference in the prevalence of hypertension between the two studies, which may be related to a difference in the recruitment of the study population, may explained this result (individuals were aged 45–70 years in the HYENA study, while participants in the DEBATS study were aged 18–90). Separated analyses of the HYENA and DEBATS datasets were also conducted to disentangle the limitations related to the few differences in setting and assessments in the two studies, but the results remained similar (see supplementary Tables S1-S4.docx).

The cross-sectional design of this study does not allow us to determine the direction of the associations observed in this paper. Individuals who are highly annoyed or highly sensitive to noise may be more likely to use medication, but it is also possible that individuals in poor health may be more likely to be annoyed or sensitive to noise and then may be more likely to attribute their symptoms to noise [62]. However, the methodology adopted for this study did not enable this question to be answered and it will need to be addressed in future studies.

## Conclusion

This study was the first to investigate the role of annoyance due to aircraft noise and of sensitivity to noise in the association between aircraft noise exposure and medication use, with such a large European study population. The results showed significant associations between aircraft noise annoyance and the use of antihypertensive, anxiolytic-hypnotic-sedative, and antasthmatic medications, as well as between aircraft noise exposure and antihypertensive medication use. They also showed an increased gradient for the use of antihypertensive, anxiolytic-hypnotic-sedative and antidepressant medications with higher levels of noise sensitivity, although the gradient for the use of antihypertensive medication did not hold for all countries. In addition, the results suggested a mediating role of aircraft noise annoyance and a modifying role of both aircraft noise annoyance and noise sensitivity in the association between noise levels and medication use. Specifically, the association between aircraft noise levels and antihypertensive medication were significantly higher in highly sensitive and in highly annoyed participants. Thus, future studies of the health effects of noise exposure have to consider both noise annoyance and noise sensitivity, in particular by using appropriate statistical models related to causal inference. Finally, the results of this study could help to recommend the implementation of appropriate measures to reduce exposure to aircraft noise, especially at night, and more particularly noise exposure of people who are noise sensitive or annoyed by aircraft noise.

## Supplementary Information

**Additional file 1:**. Supplementary Tables.

**Additional file 2:** Questionnaires.

## Data Availability

The data that support the findings of this study are not publicly available because they are covered by local agreements concerning the ethical use of data and the protection of confidentiality of individuals in all partner countries. Data are however available from the authors upon reasonable request and with permission of all national authorities. Please contact the corresponding author with permission from all national authorities. Complete versions of the questionnaire are available from the authors.

## Abbreviations

95% CI: 95% confidence interval
AIC: Akaike information criterion
ANCON: Aircraft Noise Contour Model
ATC: Anatomical Therapeutic Chemical
BMI: Body Mass Index
BP: Blood pressure
DEBATS: Discussion on the health effects of aircraft noise
FR: France
GE: Germany
GIS: Geographical Information Systems
GR: Greece
HYENA: HYpertension and Exposure to Noise near Airports
ICBEN: International Commission on the Biological Effects of Noise
ISO: International Organization for Standardization
INM: Integrated Noise Model
IT: Italy
NL: The Netherlands
OR: Odds-ratio
SW: Sweden
UK: United Kingdom
WHO: World Health Organization
