# Supplementary material for: The role of aircraft noise annoyance and noise sensitivity in the association between aircraft noise levels and medication use: results of a pooled-analysis from seven European countries
Source: BMC Public Health. 2021 Feb 5;21:300. doi: 10.1186/s12889-021-10280-3 (PMC7866660; doi:10.1186/s12889-021-10280-3)
Supplement: Supplementary file 1 — Additional file 1:. Supplementary Tables. [file 12889_2021_10280_MOESM1_ESM.docx]

**Supplementary Table S1.** **Odds ratios for medication use in relation to a 10dB(A)-increase in aircraft noise exposure at night (L_night_) and/or aircraft noise annoyance or noise sensitivity in the HYENA study**

|  |  |  | **Antihypertensive medication** | | **Antacids** | | **Anxiolytics** | | **Hypnotics** | | **Anxiolytics, hypnotics and sedatives** | | **Antidepressants** | | **Antasthmatics** | |
| --- | --- | --- | --- | --- | --- | --- | --- | --- | --- | --- | --- | --- | --- | --- | --- | --- |
|  | | | *OR* | *95%CI* | *OR* | *95%CI* | *OR* | *95%CI* | *OR* | *95%CI* | *OR* | *95%CI* | *OR* | *95%CI* | *OR* | *95%CI* |
| **M0 model** | | |  |  |  |  |  |  |  |  |  |  |  |  |  |  |
|  | **L_night_** | | **1.10** | **(1.02-1.20)** | 1.08 | (0.95-1.22) | **1.27** | **(1.01-1.59)** | 0.90 | (0.71-1.14) | 1.11 | (0.93-1.32) | 0.95 | (0.80-1.12) | 1.01 | (0.86-1.18) |
| **M1 model** | | |  |  |  |  |  |  |  |  |  |  |  |  |  |  |
|  | **Aircraft noise annoyance** | |  |  |  |  |  |  |  |  |  |  |  |  |  |  |
|  |  | *Highly vs not highly annoyed* | **1.32** | **(1.11-1.57)** | 1.09 | (0.81-1.46) | 1.47 | (0.98-2.21) | 1.58 | (0.95-2.64) | **1.59** | **(1.14-2.21)** | 1.16 | (0.79-1.70) | 1.23 | (0.87-1.73) |
| **M2 model** | | |  |  |  |  |  |  |  |  |  |  |  |  |  |  |
|  | **Noise sensitivity** | |  |  |  |  |  |  |  |  |  |  |  |  |  |  |
|  |  | *Medium vs low* | 1.07 | (0.90-1.28) | 0.96 | (0.71-1.30) | **1.80** | **(1.10-2.96)** | **1.27** | **(0.69-2.33)** | **1.57** | **(1.06-2.32)** | **1.92** | **(1.25-2.93)** | 0.92 | (0.65-1.30) |
|  |  | *High vs low* | **1.25** | **(1.05-1.47)** | 1.30 | (0.99-1.71) | **2.63** | **(1.68-4.10)** | **3.05** | **(1.84-5.04)** | **2.76** | **(1.95-3.91)** | **3.42** | **(2.33-5.02)** | 0.94 | (0.67-1.32) |
| **M3 model** | | |  |  |  |  |  |  |  |  |  |  |  |  |  |  |
|  | **L_night_** | | 1.07 | (0.98-1.17) | 1.07 | (0.94-1.22) | 1.22 | (0.96-1.54) | 0.84 | (0.65-1.08) | 1.05 | (0.88-1.25) | 0.93 | (0.78-1.10) | 0.98 | (0.83-1.15) |
|  | **Aircraft noise annoyance** | |  |  |  |  |  |  |  |  |  |  |  |  |  |  |
|  |  | *Highly vs not highly annoyed* | **1.27** | **(1.06-1.52)** | 1.05 | (0.77-1.42) | 1.34 | (0.88-2.04) | **1.77** | **(1.03-3.03)** | **1.55** | **(1.10-2.18)** | 1.19 | (0.80-1.77) | 1.24 | (0.87-1.78) |
| **M4 model** | | |  |  |  |  |  |  |  |  |  |  |  |  |  |  |
|  | **L_night_** | | **1.10** | **(1.01-1.20)** | 1.07 | (0.94-1.22) | 1.23 | (0.98-1.55) | 0.90 | (0.70-1.14) | 1.09 | (0.92-1.29) | 0.94 | (0.80-1.12) | 1.01 | (0.86-1.17) |
|  | **Noise sensitivity** | |  |  |  |  |  |  |  |  |  |  |  |  |  |  |
|  |  | *Medium vs low* | 1.07 | (0.89-1.27) | 0.96 | (0.71-1.30) | **1.77** | **(1.08-2.91)** | 1.28 | (0.70-2.34) | **1.56** | **(1.05-2.31)** | **1.93** | **(1.26-2.95)** | 0.92 | (0.65-1.30) |
|  |  | *High vs low* | **1.24** | **(1.05-1.47)** | 1.30 | (0.98-1.71) | **2.57** | **(1.65-4.03)** | **3.05** | **(1.84-5.04)** | **2.75** | **(1.94-3.89)** | **3.39** | **(2.31-4.99)** | 0.94 | (0.67-1.32) |

Models were adjusted for age, gender, education, physical activity, BMI, alcohol consumption, smoking habits and country.

**Supplementary Table S2. Odds ratios for medication use in relation to a 10dB(A)-increase in aircraft noise exposure at night (L_night_) and/or aircraft noise annoyance or noise sensitivity in the DEBATS study**

|  |  |  | **Antihypertensive medication** | | **Antacids** | | **Anxiolytics** | | **Hypnotics** | | **Anxiolytics, hypnotics and sedatives** | | **Antidepressants** | | **Antasthmatics** | |
| --- | --- | --- | --- | --- | --- | --- | --- | --- | --- | --- | --- | --- | --- | --- | --- | --- |
|  | | | *OR* | *95%CI* | *OR* | *95%CI* | *OR* | *95%CI* | *OR* | *95%CI* | *OR* | *95%CI* | *OR* | *95%CI* | *OR* | *95%CI* |
| **M0 model** | | |  |  |  |  |  |  |  |  |  |  |  |  |  |  |
|  | **L_night_** | | 1.06 | (0.80-1.40) | 0.67 | (0.45-1.00) | 0.93 | (0.63-1.37) | 0.86 | (0.54-1.39) | 0.87 | (0.64-1.20) | 0.66 | (0.40-1.08) | 1.09 | (0.65-1.81) |
| **M1 model** | | |  |  |  |  |  |  |  |  |  |  |  |  |  |  |
|  | **Aircraft noise annoyance** | |  |  |  |  |  |  |  |  |  |  |  |  |  |  |
|  |  | *Highly vs not highly annoyed* | 1.42 | (0.94-2.14) | 1.75 | (1.00-3.05) | 1.63 | (0.94-2.80) | 1.76 | (0.92-3.36) | **1.67** | **(1.06-2.62)** | 0.60 | (0.24-1.47) | **2.78** | **(1.44-5.35)** |
| **M2 model** | | |  |  |  |  |  |  |  |  |  |  |  |  |  |  |
|  | **Noise sensitivity** | |  |  |  |  |  |  |  |  |  |  |  |  |  |  |
|  |  | *Medium vs low* | 1.11 | (0.70-1.74) | 1.50 | (0.75-3.01) | 1.41 | (0.73-2.74) | **4.74** | **(1.40-16.0)** | **2.14** | **(1.16-3.97)** | 1.44 | (0.61-3.40) | 0.62 | (0.30-1.28) |
|  |  | *High vs low* | **1.83** | **(1.12-3.01)** | 1.45 | (0.67-3.11) | 1.68 | (0.82-3.44) | **6.09** | **(1.74-21.3)** | **2.90** | **(1.52-5.54)** | 1.58 | (0.64-3.92) | 0.43 | (0.18-1.02) |
| **M3 model** | | |  |  |  |  |  |  |  |  |  |  |  |  |  |  |
|  | **L_night_** | | 1.01 | (0.75-1.34) | **0.59** | **(0.39-0.90)** | 0.85 | (0.57-1.27) | 0.77 | (0.47-1.26) | 0.79 | (0.57-1.10) | 0.69 | (0.42-1.15) | 0.90 | (0.53-1.52) |
|  | **Aircraft noise annoyance** | |  |  |  |  |  |  |  |  |  |  |  |  |  |  |
|  |  | *Highly vs not highly annoyed* | 1.41 | (0.92-2.16) | **2.10** | **(1.18-3.76)** | 1.72 | (0.98-3.01) | 1.92 | (0.98-3.76) | **1.81** | **(1.13-2.89)** | 0.69 | (0.27-1.73) | **2.89** | **(1.46-5.72)** |
| **M4 model** | | |  |  |  |  |  |  |  |  |  |  |  |  |  |  |
|  | **L_night_** | | 1.08 | (0.81-1.43) | **0.66** | **(0.44-0.99)** | 0.94 | (0.63-1.39) | 0.92 | (0.57-1.48) | 0.90 | (0.65-1.24) | 0.67 | (0.41-1.10) | 1.05 | (0.63-1.76) |
|  | **Noise sensitivity** | |  |  |  |  |  |  |  |  |  |  |  |  |  |  |
|  |  | *Medium vs low* | 1.11 | (0.71-1.75) | 1.41 | (0.70-2.83) | 1.40 | (0.72-2.72) | **4.69** | **(1.38-15.9)** | **2.11** | **(1.14-3.92)** | 1.35 | (0.56-3.22) | 0.63 | (0.30-1.29) |
|  |  | *High vs low* | **1.85** | **(1.12-3.03)** | 1.37 | (0.63-2.96) | 1.66 | (0.81-3.42) | **6.03** | **(1.72-21.1)** | **2.86** | **(1.50-5.47)** | 1.51 | (0.61-3.77) | 0.43 | (0.18-1.03) |

Models were adjusted for age, gender, education, physical activity, BMI, alcohol consumption, smoking habits and country.

**Supplementary Table S3. Odds ratios (OR) for medication use in relation to a 10dB(A)-increase in aircraft noise exposure at night (L_night_) according to the level of annoyance and noise sensitivity in the HYENA study**

|  |  |  | **Antihypertensive medication** | | **Antacids** | | **Anxiolytics** | | **Hypnotics** | | **Anxiolytics, hypnotics and sedatives** | | **Antidepressants** | | **Antasthmatics** | |
| --- | --- | --- | --- | --- | --- | --- | --- | --- | --- | --- | --- | --- | --- | --- | --- | --- |
|  | | | OR | *95%CI* | OR | *95%CI* | OR | *95%CI* | OR | *95%CI* | OR | *95%CI* | OR | *95%CI* | OR | *95%CI* |
| **Aircraft noise levels at night (L_night_)^a^** | | |  |  |  |  |  |  |  |  |  |  |  |  |  |  |
|  | For not highly annoyed people | | 1.04 | (0.95-1.14) | 1.03 | (0.89-1.19) | 1.17 | (0.90-1.51) | **0.72** | **(0.54-0.96)** | 0.96 | (0.79-1.17) | 0.93 | (0.78-1.12) | 0.96 | (0.80-1.14) |
|  | For highly annoyed people | | **1.28** | **(1.05-1.56)** | 1.34 | (0.98-1.84) | 1.44 | (0.88-2.33) | 1.53 | (0.88-2.65) | **1.49** | **(1.03-2.15)** | 0.90 | (0.60-1.34) | 1.12 | (0.77-1.61) |
|  | *p_interaction_* | | ***0.05*** | | *0.12* | | *0.44* | | ***0.01*** | | ***0.03*** | | *0.85* | | *0.44* | |
| **Aircraft noise levels at night (L_night_)^b^** | | |  |  |  |  |  |  |  |  |  |  |  |  |  |  |
|  | For low noise sensitivity | | 0.94 | (0.83-1.07) | 1.02 | (0.83-1.25) | 1.43 | (0.94-2.17) | 0.76 | (0.48-1.21) | 1.05 | (0.76-1.44) | 1.06 | (0.77-1.48) | 0.83 | (0.66-1.06) |
|  | For medium noise sensitivity | | 1.07 | (0.92-1.24) | 1.02 | (0.80-1.29) | 1.26 | (0.85-1.88) | 0.72 | (0.43-1.20) | 1.03 | (0.75-1.40) | 1.13 | (0.84-1.51) | 1.17 | (0.89-1.54) |
|  | For high noise sensitivity | | **1.35** | **(1.18-1.54)** | 1.17 | (0.96-1.44) | 1.12 | (0.81-1.53) | 1.07 | (0.77-1.47) | 1.15 | (0.91-1.46) | 0.80 | (0.63-1.01) | 1.10 | (0.85-1.43) |
|  | *p_interaction_* | | ***<0.01*** | | *0.52* | | *0.62* | | *0.30* | | *0.81* | | *0.13* | | *0.12* | |

^a^ M5 model: M0 model including the interaction between L_night_ and noise annoyance.

^b^ M6 model: M0 model including the interaction between L_night_ and noise sensitivity.

Both models were adjusted for age, gender, education, physical activity, BMI, alcohol consumption, smoking habits and country.

**Supplementary Table S4. Odds ratios (OR) for medication use in relation to a 10dB(A)-increase in aircraft noise exposure at night (L_night_) according to the level of annoyance and noise sensitivity in the DEBATS study**

|  |  |  | **Antihypertensive medication** | | **Antacids** | | **Anxiolytics** | | **Hypnotics** | | **Anxiolytics, hypnotics and sedatives** | | **Antidepressants** | | **Antasthmatics** | |
| --- | --- | --- | --- | --- | --- | --- | --- | --- | --- | --- | --- | --- | --- | --- | --- | --- |
|  | | | OR | *95%CI* | OR | *95%CI* | OR | *95%CI* | OR | *95%CI* | OR | *95%CI* | OR | *95%CI* | OR | *95%CI* |
| **Aircraft noise levels at night (L_night_)^a^** | | |  |  |  |  |  |  |  |  |  |  |  |  |  |  |
|  | For not highly annoyed people | | 0.95 | (0.69-1.31) | 0.70 | (0.44-1.13) | 0.85 | (0.54-1.33) | 1.08 | (0.61-1.92) | 0.90 | (0.62-1.31) | 0.77 | (0.45-1.32) | 0.74 | (0.40-1.38) |
|  | For highly annoyed people | | 1.28 | (0.65-2.50) | **0.37** | **(0.17-0.81)** | 0.87 | (0.38-2.02) | **0.32** | **(0.13-0.79)** | 0.51 | (0.26-1.00) | 0.36 | (0.10-1.35) | 1.48 | (0.51-4.28) |
|  | *p_interaction_* | | *0.43* | | *0.16* | | *0.95* | | ***0.02*** | | *0.14* | | *0.29* | | *0.27* | |
| **Aircraft noise levels at night (L_night_)^b^** | | |  |  |  |  |  |  |  |  |  |  |  |  |  |  |
|  | For low noise sensitivity | | 0.94 | (0.51-1.73) | 0.97 | (0.36-2.60) | 2.09 | (0.69-6.33) | 2.46 | (0.25-23.8) | 2.44 | (0.82-7.23) | 1.01 | (0.29-3.48) | 0.86 | (0.35-2.14) |
|  | For medium noise sensitivity | | 0.85 | (0.57-1.27) | 0.77 | (0.44-1.35) | 0.88 | (0.51-1.51) | 0.88 | (0.46-1.66) | 0.83 | (0.53-1.28) | **0.49** | **(0.25-0.98)** | 1.02 | (0.49-2.14) |
|  | For high noise sensitivity | | **1.69** | **(1.01-2.82)** | **0.41** | **(0.20-0.84)** | 0.73 | (0.37-1.43) | 0.85 | (0.39-1.83) | 0.77 | (0.45-1.30) | 0.89 | (0.37-2.13) | 1.49 | (0.48-4.59) |
|  | *p_interaction_* | | *0.10* | | *0.26* | | *0.27* | | *0.68* | | *0.15* | | *0.45* | | *0.76* | |

^a^ M5 model: M0 model including the interaction between L_night_ and noise annoyance.

^b^ M6 model: M0 model including the interaction between L_night_ and noise sensitivity.

Both models were adjusted for age, gender, education, physical activity, BMI, alcohol consumption, smoking habits and country.
