# Supplementary material for: The role of aircraft noise annoyance and noise sensitivity in the association between aircraft noise levels and medication use: results of a pooled-analysis from seven European countries
Source: BMC Public Health. 2021 Feb 5;21:300. doi: 10.1186/s12889-021-10280-3 (PMC7866660; doi:10.1186/s12889-021-10280-3)
Supplement: Supplementary file 2 — Additional file 2: Questionnaires. [file 12889_2021_10280_MOESM2_ESM.zip › DEBATS QuestionnaireR4.doc]

# DEBATS

**Discussion on the Health Effects of Aircraft Noise**

**CONFIDENTIAL**

**Questionnaire**

**Section A: Personal details**

1. Gender

Female	⬜

Male	⬜

2. Personal details

Surname:....................................................................................................................................

First name: ................................................................................................................................

Date of birth: ..............................................................................................................................

3. Full address

............................................................................................................................................................

............................................................................................................................................................

............................................................................................................................................................

Postcode:............................................................................................................................................

**Section B: Measurements**

4. Height

|__| m |__|__| cm

Does not want to answer	⬜

5. Weight

|__|__|__| kg

Does not want to answer	⬜

**Section C: Annoyance and noise sensitivity**

6. Thinking about the last 12 months, when you are here at home, how much does aircraft noise bother, disturb or annoy you?

Extremely	⬜

Very	⬜

Moderately	⬜

Slightly	⬜

Not at all	⬜

7. Regarding noise in general, compared to people around you, do you think that you are:

Much less sensitive than people around you	⬜

Less sensitive than people around you	⬜

As sensitive as people around you	⬜

A little more sensitive than people around you	⬜

More sensitive than people around you	⬜

Don't know	⬜

**Section D : Health**

8. In the past 12 months, have you taken any medication because you were worried/anxious?

Yes	⬜

No	⬜

Do not know	⬜

Not concerned	⬜

*If yes, please specify:*

| **Name of medication** | ___________  Do not know  ⬜ | ___________  Do not know  ⬜ | ___________  Do not know  ⬜ | ___________  Do not know  ⬜ | ___________  Do not know  ⬜ |
| --- | --- | --- | --- | --- | --- |
| **Was it prescribed by a medical doctor?** | Yes ⬜  No ⬜  Do not know  ⬜ | Yes ⬜  No ⬜  Do not know  ⬜ | Yes ⬜  No ⬜  Do not know  ⬜ | Yes ⬜  No ⬜  Do not know  ⬜ | Yes ⬜  No ⬜  Do not know  ⬜ |

9. In the past 12 months, have you taken any medication because you were sad/depressed?

Yes	⬜

No	⬜

Do not know	⬜

Not concerned	⬜

*If yes, please specify*:

| **Name of medication** | ___________  Do not know  ⬜ | ___________  Do not know  ⬜ | ___________  Do not know  ⬜ | ___________  Do not know  ⬜ | ___________  Do not know  ⬜ |
| --- | --- | --- | --- | --- | --- |
| **Was it prescribed by a medical doctor?** | Yes ⬜  No ⬜  Do not know  ⬜ | Yes ⬜  No ⬜  Do not know  ⬜ | Yes ⬜  No ⬜  Do not know  ⬜ | Yes ⬜  No ⬜  Do not know  ⬜ | Yes ⬜  No ⬜  Do not know  ⬜ |

10. In the past 12 months, have you taken any medication to treat hypertension?

Yes	⬜

No	⬜

Do not know	⬜

Not concerned	⬜

If yes, please specify:

| **Name of medication** | ___________  Do not know  ⬜ | ___________  Do not know  ⬜ | ___________  Do not know  ⬜ | ___________  Do not know  ⬜ | ___________  Do not know  ⬜ |
| --- | --- | --- | --- | --- | --- |
| **Was it prescribed by a medical doctor?** | Yes ⬜  No ⬜  Do not know  ⬜ | Yes ⬜  No ⬜  Do not know  ⬜ | Yes ⬜  No ⬜  Do not know  ⬜ | Yes ⬜  No ⬜  Do not know  ⬜ | Yes ⬜  No ⬜  Do not know  ⬜ |

11. Are you currently taking any medications other than those listed above?

Yes	⬜

No	⬜

Do not know	⬜

If yes, please specify:

| **Name of medication** | ___________  Do not know  ⬜ | ___________  Do not know  ⬜ | ___________  Do not know  ⬜ | ___________  Do not know  ⬜ | ___________  Do not know  ⬜ |
| --- | --- | --- | --- | --- | --- |
| **Was it prescribed by a medical doctor?** | Yes ⬜  No ⬜  Do not know  ⬜ | Yes ⬜  No ⬜  Do not know  ⬜ | Yes ⬜  No ⬜  Do not know  ⬜ | Yes ⬜  No ⬜  Do not know  ⬜ | Yes ⬜  No ⬜  Do not know  ⬜ |

**Section E: Habits**

12. Do you usually practice a sport?

Yes	⬜

No	⬜

13. If you practice a sport, specify how many hours per day, week or month?

|__|__| hours |__|__| minutes per day

OR |__|__| hours |__|__| minutes per week

OR |__|__| hours |__|__| minutes per month

Do not know	⬜

Not concerned	⬜

14. Smoking: Are you currently:

Never smoker?	⬜

Ex-smoker?	⬜

Occasional smoker?	⬜

Daily smoker?	⬜

15. Alcohol consumption

15.1. In the past week, have you consumed any wine?

Yes	⬜

No	⬜

Do not know	⬜

15.2. If you have consumed wine, what is the maximum amount per day?

1 glass	⬜

2 glasses	⬜

3 glasses	⬜

4 glasses	⬜

5 glasses and more	⬜

1 liter and more	⬜

2 liters and more	⬜

Do not know	⬜

Not concerned	⬜

15.3. If you have consumed wine, specify how many days of the week you drank wine? (from 1 to 7 days)

Please specify: |__| days

Do not know	⬜

Not concerned	⬜

15.4. In the past week, have you had any beer or cider?

Yes	⬜

No	⬜

Do not know	⬜

15.5. If you have drunk beer or cider, what is the maximum amount per day? (in halves or large glasses)

1 Half a beer or a large glass	⬜

2 Half beers or large glasses	⬜

3 Half beers or large glasses	⬜

4 Half beers or large glasses	⬜

5 Half beers or large glasses	⬜

Do not know	⬜

Not concerned	⬜

15.6. If you drank beer or cider, indicate how many days of the week you drank beer or cider (from 1 to 7 days)

Please specify: |__| days

Do not know	⬜

Not concerned	⬜

15.7. During the last week, have you had at least one aperitif or digestive drink?

Yes	⬜

No	⬜

Do not know	⬜

15.8. If you have consumed aperitifs or digestives, what is the maximum amount per day?

1 glass	⬜

2 glasses	⬜

3 glasses and more	⬜

Do not know	⬜

Not concerned	⬜

15.9. If you have consumed aperitifs or digestives, specify how many days during the week you drank at least one aperitif or digestif (from 1 to 7 days)

Please specify: |__| days

Do not know	⬜

Not concerned	⬜

**Section F: Education**

16. What is the highest degree you have ever obtained?

No diploma	⬜

Elementary School Certificate, CAP, College Certificate, BEPC, BEP	⬜

Technical / Professional / General Baccalaureate	⬜

Baccalaureate + 2 years of higher education	⬜

Diploma higher than baccalaureate + 2 years of higher education	⬜

No diploma	⬜

*If other diploma, please specify*:.................................................................................................
