# Supplementary material for: The role of aircraft noise annoyance and noise sensitivity in the association between aircraft noise levels and medication use: results of a pooled-analysis from seven European countries
Source: BMC Public Health. 2021 Feb 5;21:300. doi: 10.1186/s12889-021-10280-3 (PMC7866660; doi:10.1186/s12889-021-10280-3)
Supplement: Supplementary file 2 — Additional file 2: Questionnaires. [file 12889_2021_10280_MOESM2_ESM.zip › HYENA QuestionnaireR4.doc]

# ID NO

#### AIRPORTS AND HEALTH STUDY

#### Questionnaire

#### C O N F I D E N T I A L

**SECTION A**

#### Personal details

**First or Christian name:**

_____________________________________________

**Surname:**

**______________________________________________**

**Date of birth:**

**______________________________________________**

| **MALE** | **FEMALE** |
| --- | --- |

**Full address:**

**______________________________________________________________**

**______________________________________________________________**

**Postcode:**

**__________________**


# ID NO

SECTION B

#### Measurements

1. **Height of participant**	*(To nearest centimetre)*
2. **WEIGHT MEASUREMENT**

**Kg (***To nearest Kilogram)***_________________________________________**

SECTION C

#### Health

3. **Please list all prescribed medications that you have taken during the last two weeks.**

(Please include prescribed sleeping pills, sedatives, tranquillizers, anti-depressants and any prescribed anti-smoking remedies as well as regular medication that you are CURRENTLY taking for a specific condition. If you can’t remember them all, please answer as best you can)

NAME OF MEDICATION

**1)____________________________________________________________**

**2)____________________________________________________________**

**3)___________________________________________________________**

**4)____________________________________________________________**

4. **Please list all ‘over the counter’ medications that you have taken over the last two weeks.**

(Please include painkillers, sleeping tablets, sedatives, anti-smoking remedies and any homeopathic or aromatherapy products that you may have purchased from your pharmacist, chemist or supermarket. If you can’t remember them all, please answer as best you can.)

NAME OF MEDICATION

**1)____________________________________________________________**

**2)____________________________________________________________**

**3)____________________________________________________________**

**4)____________________________________________________________**

**5)____________________________________________________________**

SECTION D

##### Lifestyle/Smoking/Exercise

5. **How often do you participate in leisure exercise for at least 30 minutes per session?**

(Please put a tick  in the appropriate box[es])

|  | **Moderate exercise such as brisk walking, hard gardening work, swimming, tennis, etc** | **🗸** | **Vigorous exercise such as a workout at the gym, running, speed cycling etc** | **🗸** |
| --- | --- | --- | --- | --- |
| **a) Never** |  |  |  |  |
| **b) Not regularly** |  |  |  |  |
| **c) 1-3 times a month** |  |  |  |  |
| **d) 1-3 times a week** |  |  |  |  |
| **e) 4-6 times a week** |  |  |  |  |
| **f) Every day** |  |  |  |  |
| **g) Other** *(please specify)* |  |  |  |  |

6. **Have you EVER smoked cigarettes, cigars or a pipe for as long as a year?**

(Yes means at least 1 cigarette per day or one cigar or pipe a week for 1 year)

| **YES** | NO |
| --- | --- |

| a) Do you now smoke? *(As of 1 month ago)* | YES | NO |
| --- | --- | --- |
| b) If NO to a) above, how old were you when you stopped smoking? | STOPPED AT AGE: | |
| c) On average how much do you smoke or did you smoke?  NUMBER OF CIGARETTES PER DAY |  | |
| d) On average how much do you smoke or did you smoke?  NUMBER OF PIPES PER DAY |  | |
| e) On average how much do you smoke or did you smoke?  NUMBER OF CIGARS PER DAY |  | |
| f) On average how much do you smoke or did you smoke?  NUMBER OF PACKETS OF SNUFF PER DAY |  | |

SECTION E

##### Diet

7. **On average, how many units of alcohol do you drink in a week?**

(One unit is equivalent to one glass of wine half a pint of beer or one measure of a spirit. Please enter amount in one box only

| **UNITS PER DAY** | **UNITS PER WEEK** |
| --- | --- |
|  |  |

SECTION F

**Work and Education**

8. **How many years in total have you spent in full time education?**

(Including primary, secondary, and University education but not work training)

**________________YEARS**

## **SECTION G**

## **Disturbance**

Next is a zero to ten option scale for how much noise from air, road and rail traffic bothers, disturbs or annoys you when you are here at home. If you are not at all annoyed, choose zero. If you are extremely annoyed, choose ten. If you are somewhat in between, choose a number between zero and ten.

9. **Thinking about the last 12 months or so, when you are here AT HOME, what number from zero to ten best shows how much you are bothered, disturbed or annoyed by the following potential noise sources in the daytime?**

| **NOISE FROM** | **0** | **1** | **2** | **3** | **4** | **5** | **6** | **7** | **8** | **9** | **10** |
| --- | --- | --- | --- | --- | --- | --- | --- | --- | --- | --- | --- |
| **a) ROAD TRAFFIC** | **0** | **1** | **2** | **3** | **4** | **5** | **6** | **7** | **8** | **9** | **10** |
| **b) TRAMS** | **0** | **1** | **2** | **3** | **4** | **5** | **6** | **7** | **8** | **9** | **10** |
| **c) RAILWAYS: TRAINS/TUBES** | **0** | **1** | **2** | **3** | **4** | **5** | **6** | **7** | **8** | **9** | **10** |
| **d) AIRCRAFT** | **0** | **1** | **2** | **3** | **4** | **5** | **6** | **7** | **8** | **9** | **10** |
| **e) MOTOR CYCLES** | **0** | **1** | **2** | **3** | **4** | **5** | **6** | **7** | **8** | **9** | **10** |
| **f) CONSTRUCTION** | **0** | **1** | **2** | **3** | **4** | **5** | **6** | **7** | **8** | **9** | **10** |
| **g) INDUSTRY/COMMERCIAL ACTIVITIES** | **0** | **1** | **2** | **3** | **4** | **5** | **6** | **7** | **8** | **9** | **10** |
| **h) NEIGHBOURS** | **0** | **1** | **2** | **3** | **4** | **5** | **6** | **7** | **8** | **9** | **10** |
| **i) SHOPS/RESTAURANTS/BARS** | **0** | **1** | **2** | **3** | **4** | **5** | **6** | **7** | **8** | **9** | **10** |
| **j) ANY OTHER OUTDOOR NOISE SOURCES.** *(Please specify and score for each)* | **0** | **1** | **2** | **3** | **4** | **5** | **6** | **7** | **8** | **9** | **10** |
| **k) PERMANENT INDOOR INSTALLATIONS SUCH AS VENTILATION FANS, HEATING, PLUMBING ETC.** | **0** | **1** | **2** | **3** | **4** | **5** | **6** | **7** | **8** | **9** | **10** |
| **l) ANY OTHER INDOOR NOISE SOURCES.** *(Please specify and score for each)* | **0** | **1** | **2** | **3** | **4** | **5** | **6** | **7** | **8** | **9** | **10** |

(The following question relates to NIGHT TIME noise levels experienced in THE HOME)

10. **Thinking about the last 12 months or so when you are AT HOME, what number from zero to ten best shows how much you are bothered, disturbed or annoyed by the following potential noise sources in the night-time?**

| **NOISE FROM** | **0** | **1** | **2** | **3** | **4** | **5** | **6** | **7** | **8** | **9** | **10** |
| --- | --- | --- | --- | --- | --- | --- | --- | --- | --- | --- | --- |
| **a) ROAD TRAFFIC** | **0** | **1** | **2** | **3** | **4** | **5** | **6** | **7** | **8** | **9** | **10** |
| **b) TRAMS** | **0** | **1** | **2** | **3** | **4** | **5** | **6** | **7** | **8** | **9** | **10** |
| **c) RAILWAYS: TRAINS/TUBES** | **0** | **1** | **2** | **3** | **4** | **5** | **6** | **7** | **8** | **9** | **10** |
| **d) AIRCRAFT** | **0** | **1** | **2** | **3** | **4** | **5** | **6** | **7** | **8** | **9** | **10** |
| **e) MOTOR CYCLES** | **0** | **1** | **2** | **3** | **4** | **5** | **6** | **7** | **8** | **9** | **10** |
| **f) CONSTRUCTION** | **0** | **1** | **2** | **3** | **4** | **5** | **6** | **7** | **8** | **9** | **10** |
| **g) INDUSTRY/COMMERCIAL ACTIVITIES** | **0** | **1** | **2** | **3** | **4** | **5** | **6** | **7** | **8** | **9** | **10** |
| **h) NEIGHBOURS** | **0** | **1** | **2** | **3** | **4** | **5** | **6** | **7** | **8** | **9** | **10** |
| **i) SHOPS/RESTAURANTS/BARS** | **0** | **1** | **2** | **3** | **4** | **5** | **6** | **7** | **8** | **9** | **10** |
| **j) ANY OTHER OUTDOOR NOISE SOURCES.**  (Please specify and score for each) | **0** | **1** | **2** | **3** | **4** | **5** | **6** | **7** | **8** | **9** | **10** |
| **k) PERMANENT INDOOR INSTALLATIONS SUCH AS VENTILATION FANS, HEATING, PLUMBING ETC.** | **0** | **1** | **2** | **3** | **4** | **5** | **6** | **7** | **8** | **9** | **10** |
| **l) ANY OTHER INDOOR NOISE SOURCES.** *(Please specify and score for each)* | **0** | **1** | **2** | **3** | **4** | **5** | **6** | **7** | **8** | **9** | **10** |

Next is a 1 to 6-option scale for how much you disagree or agree with the following statements. If you disagree strongly, choose 1. If you agree strongly, choose 6. If you are somewhere in between, choose a number between 1 and 6.

**Using the 1-6 scale provided, please circle how much you agree with the following statements:**

11. **No one should mind much if someone turns up his or her stereo full blast once in a while.**

**DISAGREE STRONGLY	1—2—3—4—5—6	AGREE STRONGLY**

12. **I am easily awakened by noise.**

**DISAGREE STRONGLY	1—2—3—4—5—6	AGREE STRONGLY**

13. **I get annoyed when my neighbours are noisy.**

**DISAGREE STRONGLY	1—2—3—4—5—6	AGREE STRONGLY**

14. **I get used to most noises without much difficulty.**

**DISAGREE STRONGLY	1—2—3—4—5—6	AGREE STRONGLY**

15. **Sometimes noises get on my nerves and get me irritated.**

**DISAGREE STRONGLY	1—2—3—4—5—6	AGREE STRONGLY**

16. **Even music I normally like will bother me if I am trying to**

**concentrate.**

**DISAGREE STRONGLY	1—2—3—4—5—6	AGREE STRONGLY**

17. **I find it hard to relax in a place that is noisy.**

**DISAGREE STRONGLY	1—2—3—4—5—6	AGREE STRONGLY**

18. **I am good at concentrating no matter what is going on around me.**

**DISAGREE STRONGLY	1—2—3—4—5—6	AGREE STRONGLY**

19. **I get angry with people who make noises that keep me from falling asleep or getting work done.**

**DISAGREE STRONGLY	1—2—3—4—5—6	AGREE STRONGLY**

20. **I am sensitive to noise.**

**DISAGREE STRONGLY	1—2—3—4—5—6	AGREE STRONGLY**
